# Risk factors associated with sports related injury severity in adolescents: a prospective study over a single season of sport

**DOI:** 10.1186/s13102-026-01632-w

**Published:** 2026-04-14

**Authors:** Ryan Summersby, Niamh Sheehan, Brian Caulfield, Chris Bleakley, Mark Matthews, Natalie Duff, Peter Megyesi, Jhonie Habimana, Molly McGinley, Nathan Stoops, Sinead Holden

**Affiliations:** 1 School of Public Health, Physiotherapy and Sports Science, University College Dublin, Dublin, Ireland; 2 Faculty of Life and Health Sciences, Ulster University, Belfast, UK; 3 School of Sport, Ulster University, Belfast, UK; 4 Institute for Sport and Health, University College Dublin, Dublin, Ireland; 5 Insight Research Ireland Centre for Data Analytics, Dublin, Ireland; 6 Queen’s University Belfast, Belfast, UK

**Keywords:** Adolescents, Sport, Injury, Risk factors, Athletic exposures, Dynamic balance

## Abstract

**Background:**

There is a high prevalence of injury in adolescent sports including time loss and non-time loss related injury. This study aims to investigate the risk factors associated with sports injury severity from time loss to non-time loss injuries in adolescent sports.

**Methods:**

This was a prospective cohort study. At baseline, sports participation, demographic factors and dynamic balance using an inertial sensor instrumented Quantified Y-balance Test (QYBT) were captured. Participants were followed bi-weekly for one school year to record athletic exposures and injury status using a modified Oslo Sports Trauma Research Centre Overuse injury questionnaire.

Multilevel ordinal regression models analysed the association between injury severity and risk factors of interest including age, gender, bodyweight, body mass index, somatic maturity, athletic exposures and QYBT derived dynamic balance metrics (Normalized reach distance, jerk magnitude root mean squared, gyroscopic magnitude root mean squared and gyroscopic sample entropy).

**Results:**

Of 140 adolescents (56% female) that took part, 117 were included in analysis. 63% reported at least one injury. Higher gyroscopic values in the posteromedial reach direction had lower odds of more severe injury (Odds Ratio: 0.89; 95% Confidence Interval: 0.81–0.98). Higher weekly athletic exposures were associated with higher odds of sustaining a more severe injury (Odds Ratio: 1.11; 95% Confidence Interval: 1.04–1.18).

**Conclusions:**

Adolescents in a mixed sport population who have higher athletic exposures and poorer dynamic balance performance as assessed using the QYBT are at higher risk of more severe injury. Dynamic balance and athletic exposures represent trackable and modifiable factors that are associated with injury in adolescent sports.

**Supplementary Information:**

The online version contains supplementary material available at 10.1186/s13102-026-01632-w.

## Introduction

Sports participation offers many physical and mental benefits to young adolescents but comes with an inherent risk of injury [1]. The burden of adolescent sports injuries is high, with sports injury prevalence ranging from 34% to 65% among adolescent athletes identified in a recent systematic review [2]. Injury prevalence and burden of sports related injury in adolescents has traditionally focused on time loss injuries [3] but this can underestimate the injury prevalence, particularly in adolescent athletes (Summersby R, Sheehan N, Bleakley C, Caulfield B, Matthews M, Duff N, et al: Pain and Injury Burden in Adolescent Athletes: A Prospective Study, unpublished). This is partly due to a culture, wherein younger athletes are more likely to continue to play through pain, despite knowing it may worsen their injury [4].

There are a range of intrinsic (BMI, age, maturity status, dynamic balance performance) and extrinsic (Athletic exposures, sports played, injury history) risk factors associated with sports related injury [2, 5–8]. However, this evidence has been derived primarily from elite level adult athletes involved in single sports. Adolescents, particularly those involved in team sports, are underrepresented in this field of research.

In mixed sport youth cohorts, the association between training load and injury risk remains inconsistent and unclear [9]. Similarly in adult studies there is a lack of consistency in what is the most appropriate methodological frameworks to quantify training load accurately for analysis [10]. Despite heterogeneity in methods some studies have shown training load as a potential risk factor for injury within youth and adult sport [11]. Previous research on running athletes showed week to week increases of 24% or more in distance ran led to a substantial increase in risk of injury [12]. These studies have illustrated associations of sports injury with internal and external training load metrics including distance ran, minutes played per game and even the number of accelerations the athlete completed in a match [11, 12]. These measures despite being insightful are more burdensome to track and are often not possible to track particularly within a non-elite youth sports setting where resources may be limited. In a non-elite youth sports cohort the number of athletic exposures for an athlete may be a more appropriate and practical measure of external training load. In this way training load as a risk factor can be tracked but athlete feedback is also more tangible and meaningful for an adolescent athlete to understand. In a study with elite level adult and youth field hockey players, a greater number of competition athletic exposures across the hockey season was associated with higher risk of injury particularly lower limb [13]. Similarly, the Childhood Health, Activity, and Motor Performance School Study Denmark a longitudinal cohort study, showed that for children 8–15 years of age, risk of traumatic and overuse knee injuries increased by 46% to 140% when they engaged in over two athletic exposures per week [14].

Single leg dynamic balance performance is a fundamental component of many team sports e.g. rugby, basketball, soccer [15]. Impaired single leg dynamic balance performance has been shown to have a significant impact on sports related injury rates in secondary school aged athletes [16]. This impairment in dynamic balance has been attributed to sensory-motor mechanisms, where rapid skeletal growth during adolescence is coupled with a period of ‘motor awkwardness’ [17]. The Quantified Y-balance test (QYBT), which utilises an inertial measurement unit (IMU), is a valid way to objectively assess lower limb dynamic balance, beyond standard reach distance measures [18]. Previous QYBT research reported that athletes with sub-optimal dynamic balance, defined as gyroscopic sample entropy greater than or equal to 1.2 in the anterior reach direction, had a 2.81-times greater odds of sustaining a concussion [5]. To date, we are not aware of any studies linking dynamic balance performance measured using the QYBT to musculoskeletal injury in sport. This study is therefore one of the first to explore this association.

The aim of this study was to prospectively investigate the risk factors associated with sports related injury/pain in an adolescent population. Risk factors of interest were age, gender, bodyweight (BW), body mass index (BMI), somatic maturity, athletic exposures and QYBT derived dynamic balance metrics (Normalized reach distance, jerk magnitude root mean squared, gyroscopic magnitude root mean squared and gyroscopic sample entropy).

## Materials and methods

### Study design

This study was designed as a prospective cohort study among secondary school student athletes on the Island of Ireland. Enrolment and baseline assessments were conducted on a rolling basis (October 2023 to December 2023). Participants were followed for a school year (until May 2024) via a mobile phone application. The reporting of this study followed the International Olympic Committee consensus statement guidelines on Recording and Reporting of Epidemiological Data on Injury and Illness in Sport 2020, and Strengthening the Reporting of Observational Studies in Epidemiology - Sports Injury and Illness Surveillance (STROBE-SIIS) guidelines [19, 20]. Ethical approval was sought and approved by the University College Dublin Human Research Ethics Committee (reference: LS-23-28) on 27th of April 2023 for this study. Written informed parental consent and participant assent was obtained for all participants under the age of 18.

### Participants and recruitment

Participants were recruited through secondary school’s sports coaches and physical education teachers on the Island of Ireland. Potential schools were approached and given information leaflets on the study before agreeing to take part. Once a school agreed to take part, study information leaflets were provided to sports coaches and physical education teachers to distribute to potentially eligible students. To be eligible for inclusion participants had to be secondary school students aged 14–18 years of age participating in any (team/individual) field, court, or track sports on a regular basis, and could provide written informed consent and/or parental/guardian consent as appropriate. Exclusion criteria included any current injury/disability/neurological condition/medical condition/sports related pain that would impede performance on the QYBT. There were no restrictions on sports played or level of sport played. For multisport participants, their primary sport was self-identified defined as the sport played most often.

### Baseline data collection

Baseline data collection included anthropometrics, demographics and sport/injury history as well as a QYBT assessment (outlined below). Following this, participants were onboarded to the mobile phone application (WISP PRO) to capture follow-up data. The WISP PRO mobile application and the QYBT data collection software for this study was developed collaboratively for this study by University College Dublin Insight Research Ireland Centre for Data Analytics. Anthropometric measures were standing height, sitting height, bilateral leg length, and BW. These measures were then used to calculate BMI and somatic maturity using the Moore-1 predicted maturity offset formulas separately for males and females [21]. A baseline characteristics questionnaire captured gender, age (birth month and year), number and type sports played, number of self-reported injuries within the last 6 months, self-perceived level of sport seriousness, and leg dominance (defined as the leg they used to kick a ball as far as possible) [22].

**Fig. 1:**
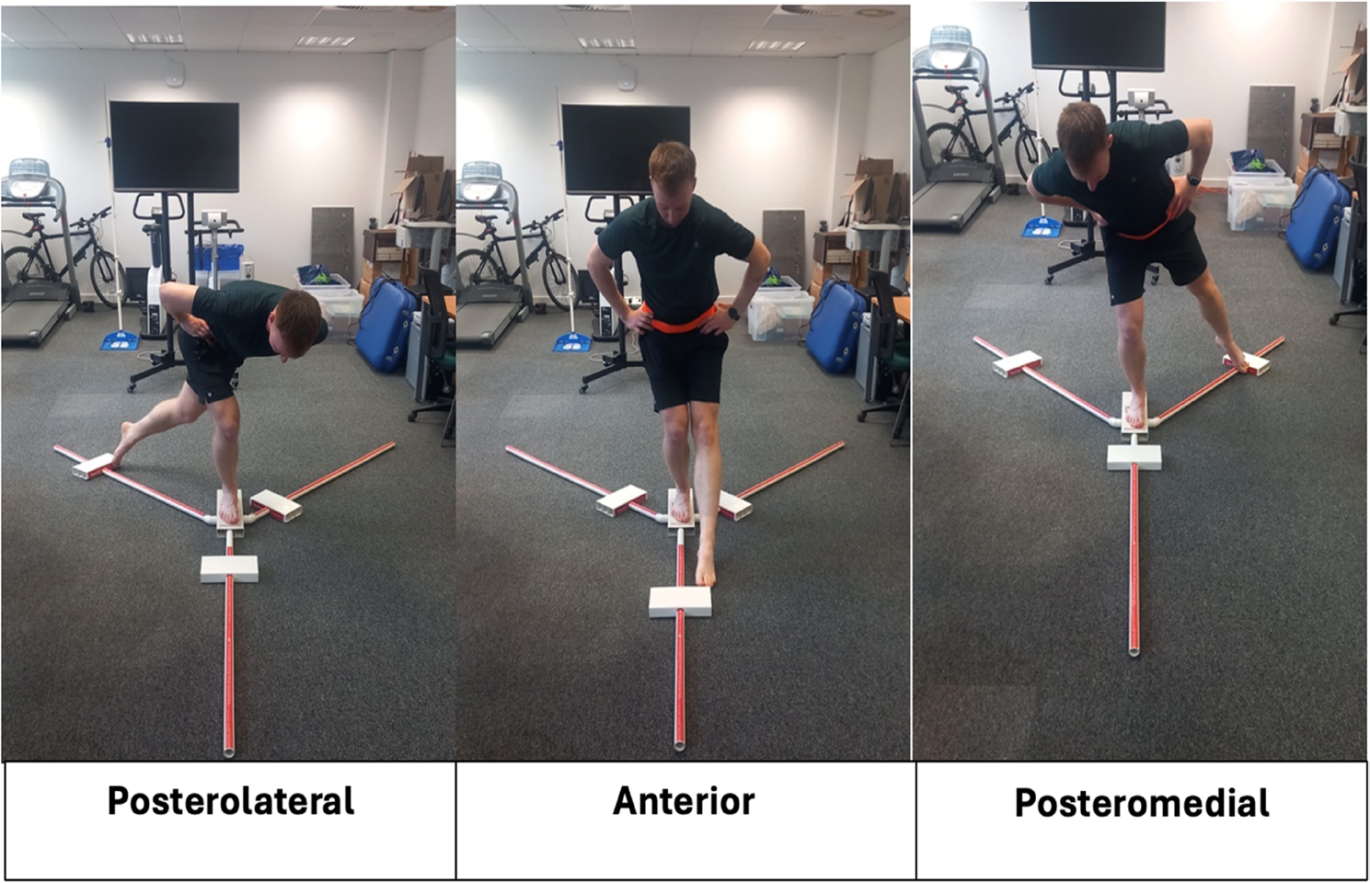
QYBT reach directions

The QYBT comprised of an inertial sensor instrumented version of the Y Balance test which is used to assess dynamic balance and is associated with risk of injury [5, 23]. QYBT testing procedures followed previously published QYBT research [5, 23]. Participants begin standing on their dominant leg, and reach as far as possible in three directions (anterior, posteromedial and posterolateral – Fig. 1) with the contralateral limb. To qualify as a successful test, participants need to return to bilateral stance without losing their balance or placing the reaching foot on the ground. Participants completed four practice trials followed by three recorded trials in each direction, with a break between each trial for the tester to reset the YBT and input reach distances into the data collection app. The dominant leg was chosen as the test leg as a proxy for overall lower limb dynamic balance performance due to time constraints in physical education classes for the participants.

**Fig. 2:**
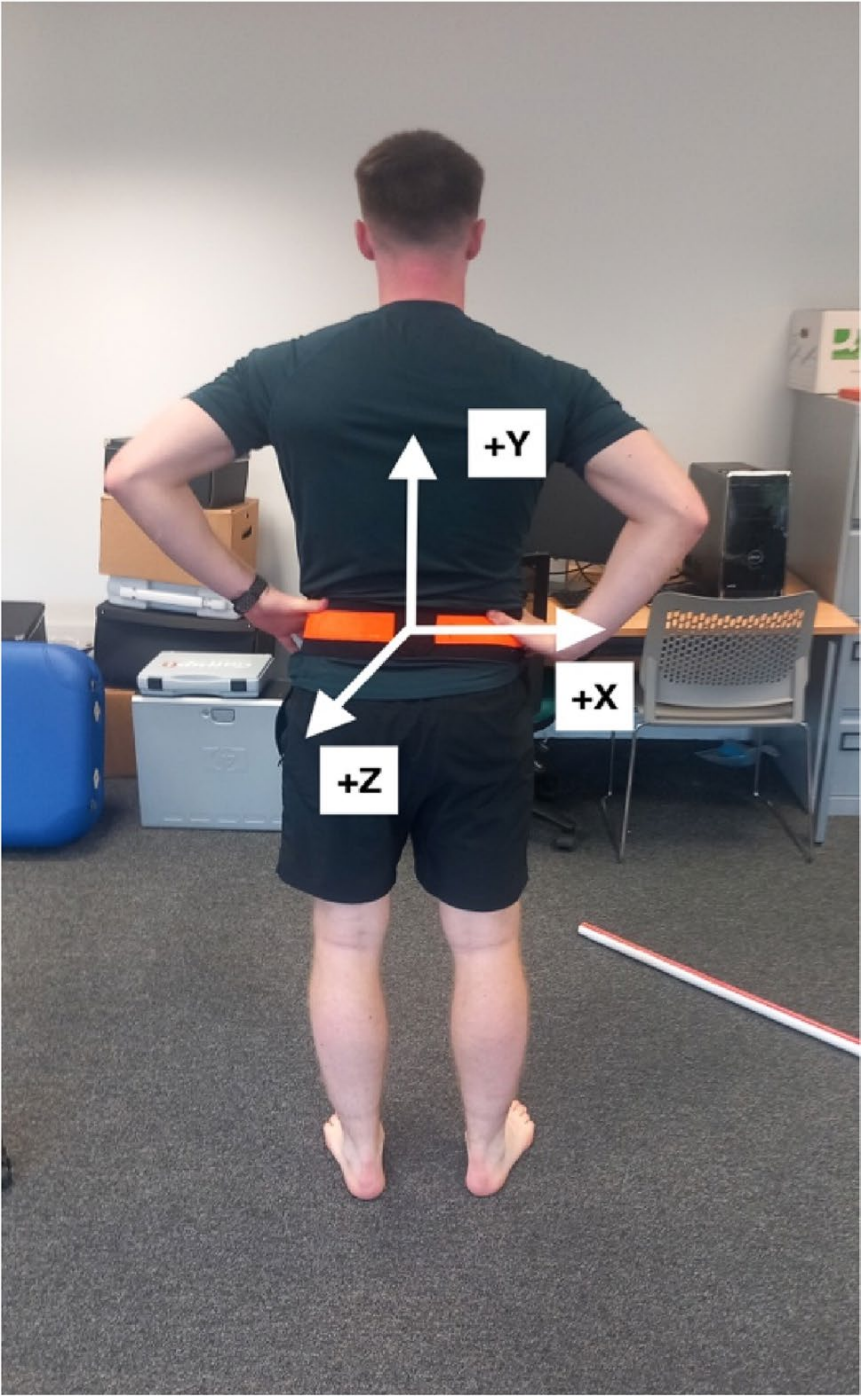
Inertial sensor mounted on the fourth lumbar vertebrae

The QYBT is completed with a single inertial sensor (Shimmer 3, Shimmer Sensing) mounted at the level of the fourth lumbar vertebrae, the approximate centre of mass (COM) (Fig. 2). The inertial sensor was connected via Bluetooth to an Android tablet which collected the inertial sensor data via a custom made WISP application programmed to collect gyroscopic data at a frequency of 51.2 Hz during each repetition. Analogue QYBT reach distances were recorded after each repetition of maximal reach, the inertial sensor captured data throughout each repetition. If a participant lost their balance, placed the reaching foot on the ground for support or stepped off the apparatus, the test was discarded and repeated again. All balance testing was completed by Chartered Physiotherapists and experienced researchers.

### Follow-up data collection

After inclusion, participants received a survey every two weeks (see Appendix 1.) through a mobile application (WISP PRO). The questionnaire consisted of a modified version of the updated Oslo Sports Trauma Research Centre Overuse Injury Questionnaire (OSTRC) [24], alongside training and competition exposures. In this context, an athletic exposure was defined as one single training or competition where one athlete is exposed to the possibility of sports injury [25]. Participants playing more than one sport reported total athletic exposures from across the various sports they played. The updated OSTRC Overuse questionnaire examines the impact of sports related injuries on; participation, pain, performance and any training/competition modification caused by the injury. Questionnaire responses are allocated a value (0 (no problem), 8 (mild), 17 (moderate) or 25 (maximum level of severity)), and these are summed giving a score from 0 to 100 [24, 26]. This is then used to calculate the overall impact/severity of this injury in the previous 14 days from 0 to 100. A score of 0 indicating no injury and 100 being maximum severity with the participant not able to partake due to sports injury.

### Data processing and analysis

Initial data cleaning of the dynamic balance variables involved visualisation of all of the inertial sensor acceleration data signals through a Python (Python 3.12) screening pipeline (designed by a Biomedical Engineer part of the research team – J.H) in order to screen for any noisy data that needed to be filtered or any outlier signal data that needed to be discarded. From this, the screened and filtered inertial signal data was then fed through a MATLAB (R2024b, MathWorks) processing pipeline (implemented by a Biomedical Engineer part of the research team – J.H) to calculate the jerk magnitude, gyroscopic magnitude and sample entropy. These are validated measures of the smoothness and complexity of the movement of each repetition of the QYBT for each participant [27]. Formulae for these variables are available in Appendix 2.

Each of the QYBT outcome measures shown below were recorded in each of the three QYBT reach directions (see Fig. 2): anterior, posteromedial and posterolateral.

*Jerk magnitude root mean squared (JERK)*: is a measure of the “constraint” of the COM during the QYBT through measurement of the overall smoothness of movement, the average rate of change in acceleration in relation to time (t) in the x, y, z axis (Fig. 2) within the IMU and is measured in meters per second cubed ($$m/{s}^{3})$$ [28, 29]. Previous research investigating the effect of previous concussion on QYBT performance in American Football and Ice Hockey players showed lower JERK values in the group with a history of concussion indicating more constraint in their COM movement [23].

*Gyroscopic magnitude root mean squared (GYRO)*: is a measure of the overall rotational movement, the average angular velocity in relation to time (t) in the x, y, z axis (Fig. 2) within the IMU. Higher values of GYRO indicate greater overall speed of rotation of the COM during the QYBT and is measured in degrees of rotation per second (degrees/s) [5, 30].

*Gyroscopic Magnitude Sample Entropy (GYRO SEN)*: is a unitless measure of the randomness and complexity of the rotational movement, the regularity or irregularity of a gyroscopic time series signal [31]. GYRO SEN has been used to differentiate between pre-concussion and post-concussion postural movement control [5] with lower GYRO SEN indicating less irregularity and complexity in their postural control or better dynamic balance performance. Higher GYRO SEN has been shown to be an indicator of poorer dynamic balance performance and greater risk of concussive injury in rugby [5].

*Normalized Reach Distances (%)*: Analogue reach distances were also normalized relative to the participants leg length.

### Statistical methods

#### Dependent variables

The dependent variable was the OSTRC injury severity categories split into four ordinal categories: No Injury > All Problems Injury > Substantial Injury > Time Loss Injury. These injury severity categories were defined by previous literature involving the OSTRC [24, 32, 33] and are summarised below:

- *No Injury***–** Any questionnaire where a participant reported full participation in sport without pain or injury (OSTRC Question 1 = A).
- *All Problems Injury*** -** A participant reporting any physical complaint in response to question one on the OSTRC (OSTRC Question 1 = B, C or D).
- *Substantial Injury*** -** Any participant reporting any physical complaint leading to moderate to severe modifications in training volume, moderate to severe reduction in performance, or the complete in ability to participate in sports due to injury (OSTRC Question 2 or 3 = C or D).
- *Time loss Injury*** -** Any participant reporting a complete inability to participate in sport (OSTRC Question 1 = D).

#### Independent variables

There were three subsets of independent variables used within an ordinal mixed effect regression analysis: Athletic exposures variable, QYBT variables, and Anthropometric and Demographic variables. All independent variables were continuous variables except for gender which was binary categorical.

- *Athletic exposures *– The two week rolling average of athletic exposures, was calculated using a one-week lag. This approach was taken to account for the reduced training load that often occurs during the week an injury occurs.
- *QYBT Variables*: JERK, GYRO, GYRO SEN and normalized reach distance were four separate QYBT variables across the three QYBT reach directions – anterior, posteromedial and posterolateral.
- *Anthropometric and Demographic Variables*: Age (years), BW (kg), gender (male=0, female=1), Moore-1 Maturity Offset (Maturity Offset - time in years since peak height velocity an indicator of somatic maturity [21]) and BMI (kg/m^2^).

Baseline demographic variables requiring processing were maturity offset, injury and gender. Maturity Offset is a continuous variable, a measure of the time in years before or after peak height velocity which is an indicator of somatic maturity status. Maturity offset was calculated using the Moore-1 predicted maturity offset formulas separately for males and females [21]. Gender is a categorical independent variable and was coded male = 0, female = 1. All participants identified as either male or female.

### Statistical analysis

For baseline demographics means and related standard deviations were calculated for continuous numerical variables (height, BW, age) along with median and interquartile ranges for categorical variables (Number of sports played and number of previous injuries) across males and females.

Ordinal mixed-effects regression models were used to assess the association between predictor variables and the ordinal OSTRC injury severity categories: No Injury > All Problems Injury > Substantial Injury > Time Loss Injury. Ordinal mixed-effects regression models were used as it allows for the addition of participants as a random effect accounting for within-subject correlation and variability between participants associated with repeated measures datasets. Goodness of fit for each model was assessed and compared using McFadden’s Pseudo R-squared (which quantifies the extent to which the model explains the variance in the outcome variable, compared to a null model with no predictor variables).

Initially all predictor variables were analysed individually in unadjusted models with participants as a random effect. Any statistically significant predictor variables (*P* < 0.05) at this stage were considered for further analysis in a multivariable model. To improve readability of results tables only statistically significant QYBT predictor variables were displayed. To avoid overfitting and multicollinearity where more than one balance variable was statistically significant in the unadjusted models, the balance variable with the highest McFadden’s Pseudo R-Squared was carried forward to the adjusted model.

For the adjusted and unadjusted models, odds ratios (OR) and 95% confidence intervals for the OR were calculated to quantify the association between predictor variables and the odds of being in a higher injury severity category. To minimise risk of overfitting and to ensure clear interpretability, the final adjusted model was limited to two predictor variables. This is due to the small sample size for analysis and to ensure focus on the most significant variables associated with injury severity. The selection of the two predictor variables included in the final model was guided by theoretical relevance of the variable, statistical significance of the variable (*p* < 0.05) and the greater McFadden’s Pseudo R-Squared value.

Predicted probabilities for each injury severity category for the predictor variables included in the final adjusted model were generated and visualized to demonstrate the relationship between the predictor variables and each of the injury severity categories.

Statistical significance for all tests and models was set at *p* < 0.05.

Statistical analysis was conducted with RStudio (V.2024) packages: stats, ordinal, rcompanion, and performance.

## Results

**Fig. 3:**
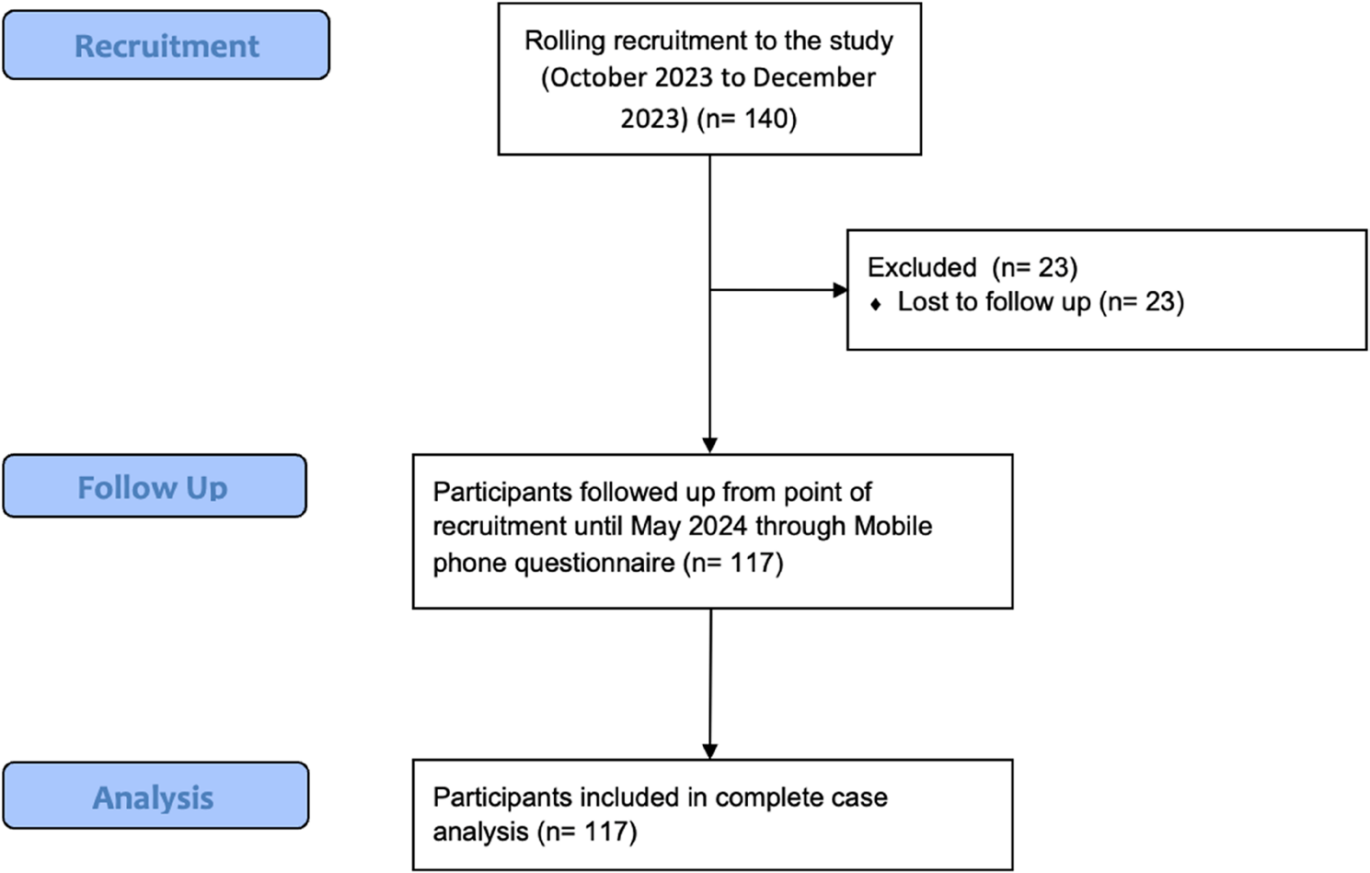
CONSORT style flow chart of participants recruited, followed up and included in the analysis [34]

A total of 140 secondary school students (78 female (56%) and 62 male (44%)) from across eight secondary schools agreed to take. Participant descriptives are outlined in Table 1. Of those 140 students, 117 (83.6%) were successfully onboarded to the app and completed at least one follow-up questionnaire and were included in analysis. Over the 32-week follow-up period the response rate was 47.5% (720/1515 questionnaires returned). The CONSORT style flow diagram is shown in Fig. 3 [34]. Throughout follow up 120 minor injuries were reported, 60 substantial injuries and 20-time loss injuries − 43 participants reported no injuries. The distributions of age across males and females were similar with a mean age of 15 years for both males and females. The five most played sports respectively were: Field hockey, rugby, Gaelic football, soccer and netball (see Table 1 for full details).

**Table 1:** Participants characteristics

Gender (*N*)	Female	Male
	64	53
Mean Age (SD) - years	15.6 (1.0)	15.3 (0.8)
Mean Stand Height (SD) - cm	166.1 (5.9)	176.3 (6.6)
Mean Sit Height (SD) - cm	85.4 (3.4)	88.2 (3.7)
Mean BMI (SD) – kg/m^2^	21.6 (3.2)	21.5 (3.0)
Mean BW (SD) - kg	59.3 (9.6)	67.1 (11.8)
Mean Maturity Offset (SD) - years	3.6 (0.8)	1.7 (0.7)
Mean Anterior Normalized Reach Distance (SD) - %	68.9 (6.8)	68.1 (6.2)
Mean Posteromedial Normalized Reach Distance (SD) – %	106.8 (8.6)	108.5 (7.8)
Mean Posterolateral Normalized Reach Distance (SD) - %	102.0 (11.3)	104.3 (7.8)
Mean Anterior Jerk (SD) - m/s^3^	0.3 (0.1)	0.4 (0.1)
Mean Posteromedial Jerk (SD) - m/s^3^	0.3 (0.1)	0.3 (0.1)
Mean Posterolateral Jerk (SD) - m/s^3^	0.3 (0.1)	0.3 (0.1)
Mean Anterior GYRO (SD) – degrees/s	12.6 (5.0)	12.6 (4.8)
Mean Posteromedial GYRO (SD) - degrees/s	15.0 (4.5)	14.5 (4.0)
Mean Posterolateral GYRO (SD) - degrees/s	17.0 (4.6)	16.6 (4.4)
Mean Anterior GYRO SEN (SD)	1.1 (0.3)	1.1 (0.3)
Mean Posteromedial GYRO SEN (SD)	0.9 (0.3)	0.9 (0.2)
Mean Posterolateral GYRO SEN (SD)	0.8 (0.3)	0.8 (0.2)
Median No. of Sports Currently Playing (IQR)	2 (1)	2 (1)
Median No. of Previous Injuries (IQR)	1 (2)	1 (1)
Primary Sport Played: N (%)		
Hockey	38 (59.4%)	3 (5.7%)
Rugby	2 (3.1%)	21 (39.6%)
Gaelic Football	6 (9.4%)	13 (24.5%)
Soccer	1 (1.6%)	9 (17%)
Netball	8 (12.5%)	0 (0%)
Basketball	0 (0%)	4 (7.5%)
Swimming	4 (6%)	1 (1.9%)
Hurling/Camogie	1 (1.6%)	0 (0%)
Athletics	1 (1.6%)	1 (1.9%)
Dance	0 (0%)	1 (1.9%)
Horse Riding	1 (1.6%)	0 (0%)
Tennis	1 (1.6%)	0 (0%)
Volleyball	1 (1.6%)	0 (0%)

All data are described using mean (SD – standard deviation), n (%) or median (IQR -Interquartile range)

*GYRO* Gyroscopic magnitude root mean squared, *JERK* Jerk magnitude root mean squared, *GYRO SEN* Gyroscopic Magnitude Sample Entropy, *BW *Body weight, *BMI *Body mass index

### Missing data

Due to sensor and data collection software failure there were some missing data points from the baseline QYBT variables and anthropometric variables BW and BMI. The percentage of missing data for each affected variable for the baseline data set is illustrated in Table 2. Across all affected variables missing data ranged from 1.7% to 5.9%. The missing data was assumed to be missing at random due to sensor and data collection software failure unrelated to participant balance performance or anthropometric measures. No imputation was done for these missing data points to avoid introducing bias within the data. Observations with missing data points were excluded where that variable was used in the analysis.

**Table 2:** Percentage of missing data for each affected variable

Variable	Baseline Missing Data (%)
Anterior Normalized Reach Distance	1.7% (2/117)
Posteromedial Normalized Reach Distance	1.7% (2/117)
Posterolateral Normalized Reach Distance	1.7% (2/117)
Anterior GYRO	5.9% (7/117)
Posteromedial GYRO	5.9% (7/117)
Posterolateral GYRO	5.9% (7/117)
Anterior JERK	5.9% (7/117)
Posteromedial JERK	5.9% (7/117)
Posterolateral JERK	5.9% (7/117)
Anterior GYRO SEN	5.9% (7/117)
Posteromedial GYRO SEN	5.9% (7/117)
Posterolateral GYRO SEN	5.9% (7/117)
BW	3.4% (4/117)
BMI	3.4% (4/117)

*GYRO* Gyroscopic magnitude root mean squared, *JERK* Jerk magnitude root mean squared, *GYRO SEN* Gyroscopic Magnitude Sample Entropy, *BW* Body weight, *BMI* Body mass index

### OSTRC injury severity category models

Unadjusted ordinal mixed effects regression models are displayed in Table 3. Balance variables; posteromedial GYRO, posterolateral JERK and posteromedial GYRO SEN showed statistically significant associations with OSTRC injury severity (*p* < 0.05).

Athletic exposures also showed a statistically significant association with increasing OSTRC injury severity with higher athletic exposures values (*p* = 0.005). The anthropometric variable BW also showed statistically significant association with increasing OSTRC injury severity with higher participant BW (*p* = 0.007).

In the final adjusted model (Table 4) posteromedial GYRO (*p* = 0.022) and athletic exposures (*p* = 0.001) show statistically significant associations with OSTRC injury severity with a McFadden’s R-squared of 0.356. The final adjusted model shows that for every degree/second increase in posteromedial GYRO the odds of being in a higher OSTRC injury severity (e.g. substantial or time loss) category compared to a lower injury category (e.g. No injury or all problems injury) is 0.89 times lower. This indicates that as GYRO values increase, the risk of severe injury decreases.

For athletic exposures the final adjusted model shows that for every one athletic exposure increase in athletic exposures each week the odds of being in a higher OSTRC injury severity (e.g. substantial or time loss) category compared to a lower injury category (e.g. No injury or all problems injury) is 1.11 times higher. This indicates that with higher athletic exposures there is increased risk of more severe injury.

**Table 3:** Unadjusted injury severity models: estimates of association of predictor variables

Unadjusted Models – Predictor Variable	OR	95% CI - Lower	95% CI - Upper	*p*-value	McFadden’s Pseudo *R*-squared	Random Effects (Variance) - Participant
Posteromedial GYRO*	0.873	0.802	0.949	0.002	0.071	1.457
Posterolateral JERK*	0.013	< 0.001	0.583	0.025	0.066	1.546
Posteromedial GYRO SEN*	2.739	2.721	2.757	< 0.001	0.064	1.608
Athletic exposures*	1.110	1.032	1.194	0.005	0.309	1.476
Age (years)	1.107	0.794	1.543	0.548	< 0.001	1.467
BW (kg)*	1.037	1.010	1.064	0.007	0.040	1.382
Maturity Offset (years)	1.018	0.792	1.307	0.890	< 0.001	1.481
Gender	0.713	0.386	1.315	0.279	0.001	1.456
BMI	1.094	0.992	1.207	0.378	0.037	1.475

*Indicates statistically significant variables (*p*-value<0.05)

*OR *Odds Ratio, *CI *Confidence Interval, *GYRO* Gyroscopic magnitude root mean squared, *JERK* Jerk magnitude root mean squared, *GYRO SEN* Gyroscopic Magnitude Sample Entropy, *BW *Body weight, *BMI *Body mass index

**Table 4:** Adjusted Injury Severity Models: Estimates of Association of Predictor Variables

Variables	OR	95% CI - Lower	95% CI - Upper	*p*-value	McFadden’s Pseudo *R*-squared	Random Effects (Variance) - Participant
Final Adjusted Model						
Athletic exposures*	1.109	1.042	1.179	0.001	0.356	1.431
Posteromedial GYRO*	0.890	0.806	0.983	0.022		

* Indicates statistically significant variables (*p*-value < 0.05)

*OR* Odds Ratio, *CI* Confidence Interval, *GYRO* Gyroscopic magnitude root mean squared, *JERK* Jerk magnitude root mean squared, *GYROSEN* Gyroscopic Magnitude Sample Entropy, *BW* Body weight, *BMI* Body mass index

### Visualisation of relationship between athletic exposures and posteromedial GYRO for each injury severity category

**Fig. 4:**
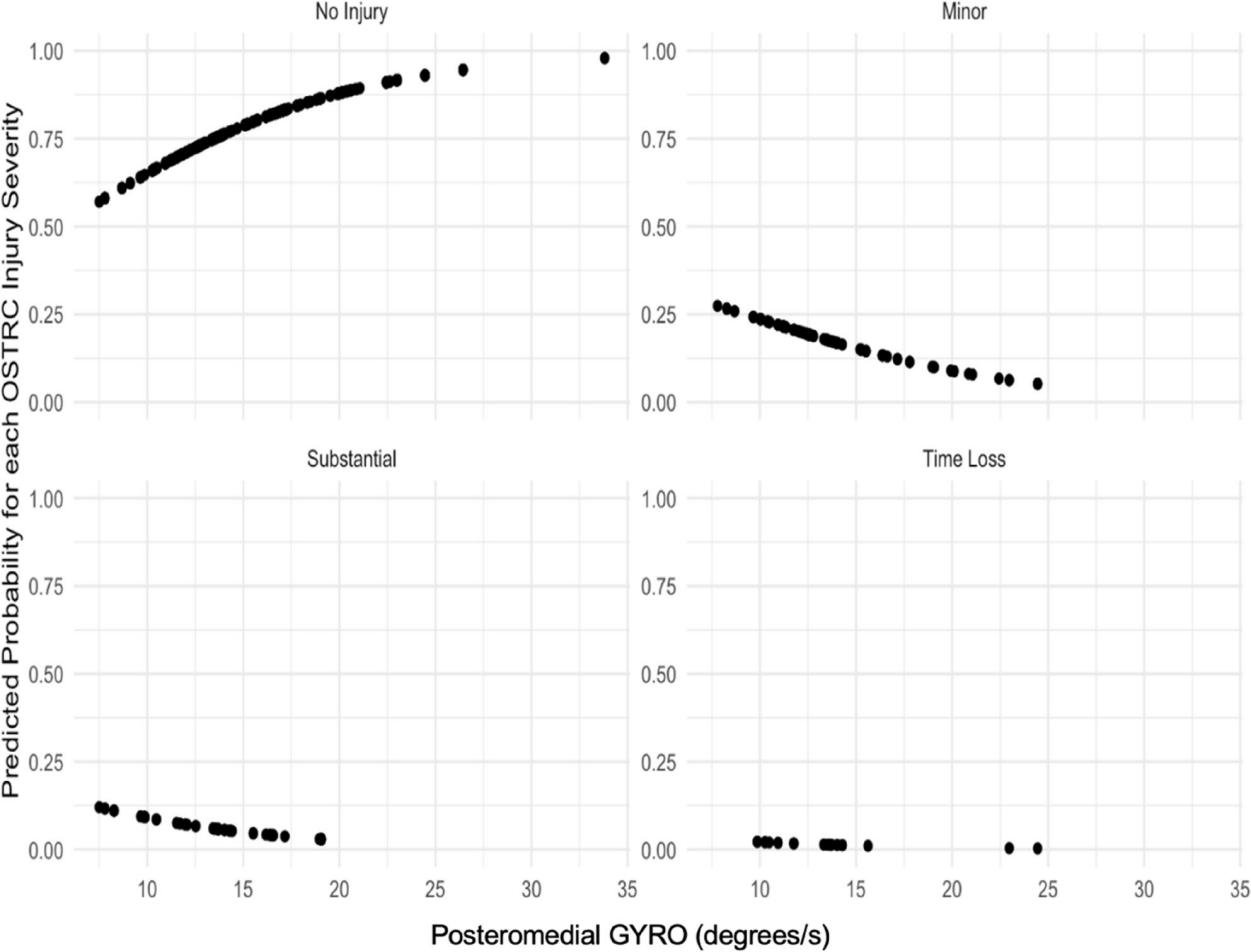
Injury Severity Category Predicted Probabilities across Posteromedial GYRO. GYRO= Gyroscopic magnitude root mean squared (degrees/s)

**Fig. 5:**
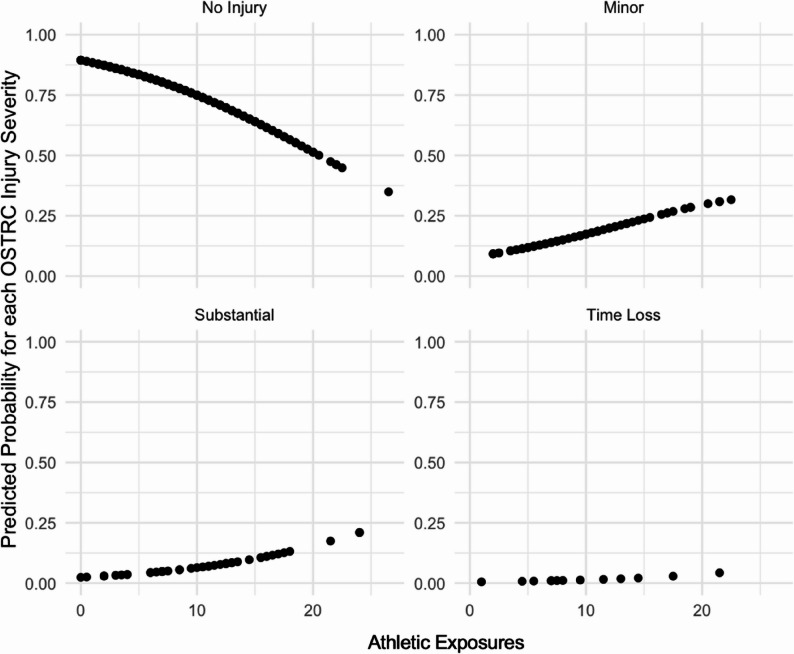
Injury Severity Category Predicted Probabilities across Athletic Exposures

Predicted probabilities for each Injury Severity Category across the Posteromedial GYRO and athletic exposures predictor variables are displayed in Figs. 4 and 5 respectively. Overall Fig. 4 shows that higher values of posteromedial GYRO increase the predicted probability of having no injury itself and decreases the predicted probability of having a more severe injury – minor and substantial injuries (*p* = 0.022). The visual relationship of posteromedial GYRO and time loss injuries is less clear due to fewer time loss injuries recorded overall. Overall Fig. 5 shows that higher numbers of athletic exposures decrease the predicted probability of having no injury itself and increases the predicted probability of having a more severe injury – minor, substantial and time loss injuries (*p* = 0.001).

## Discussion

The results from this study show that within a general mixed sport adolescent cohort on the Island of Ireland dynamic balance performance and changes in athletic exposures throughout the season are associated with injury severity. The use of ordinal regression models in sports injury research has the potential for generating valuable insights particularly in identifying risk factors associated with time loss and non-time loss injuries in sports injury research beyond simply injured and uninjured binary logistic regression analysis [35]. The ordinal mixed effects regression models (Tables 3 and 4) as a whole, indicates that lower limb dynamic balance performance assessed using the QYBT and athletic exposures are associated with OSTRC injury severity independent of potential confounding factors. Posteromedial GYRO is negatively associated with injury severity, greater posteromedial GYRO values are associated with reduced predicted injury severity. However, athletic exposures show the greatest predictive power of all of the predictor variables with McFadden’s Pseudo R-Squared value of 0.309 in the unadjusted model indicating very good model fit and that athletic exposures is a strong predictor of injury severity with greater risk of time loss and non-time loss injuries with increasing number of athletic exposures.

Posteromedial GYRO showed a statistically significant association with OSTRC injury severity, with increasing posteromedial GYRO values there was a reduced risk of more severe injury including time loss injury compared to non-time loss injury. This finding suggests that maintaining COM control at greater angular velocity magnitudes may influence injury risk during single leg dynamic balance assessment in an adolescent sporting cohort which has not been shown in the literature to date. Similarly, although not all statistically significant all JERK and GYRO measures showed that higher values of JERK and GYRO were associated with reduced predicted injury severity. A study using the QYBT in an elite adult rugby union cohort also found that poorer dynamic balance performance as measured from the gyroscopic magnitude sample entropy variable was associated with greater risk of a concussive injury [5]. This aligns with other studies showing that slower acceleration changes as measured from IMU based dynamic balance testing is associated with previous injury [5, 23]. Further research utilising IMU derived metrics like GYRO is required to understand if these associations are due to an ability of the adolescent to maintain COM control at greater magnitudes of angular velocity or this is the motor coordination response to this single leg balance task.

Furthermore, the ordinal regression analysis showed that for every one unit (degrees/second) increase in GYRO in the posteromedial direction during the QYBT the adolescent had a 0.89-lesser odds or a 11% decrease in the odds of experiencing a more substantial injury (e.g. increased risk of time loss injury compared to non-time loss injury). The GYRO variable in the posteromedial reach direction for both the injured and uninjured cohort has a median of 13.69 degrees/second and an interquartile range of 4.94 (1st Quartile = 11.53 degrees/second; 3rd Quartile = 16.47 degrees/second) indicating a potentially 0.56 times lesser odds of a more severe injury comparing those in the 1st Quartile to those in the 3rd Quartile $\left(Absolute\, \Delta odds=OR_{4.94}=0.89^{4.94}\right)$. This illustrates a 44% decrease in the odds of more severe injury for those with faster posteromedial GYRO moving from the 1st Quartile to the 3rd Quartile. This highlights a potentially strong association of posteromedial GYRO scores with injury severity in this adolescent cohort. Contrasting this for every one unit increase in athletic exposures the adolescent had an 11% increase in odds of experiencing a time loss injury compared to non-time loss substantial injury. This provides greater clarity to the association with injury of acute changes in athletic exposures in a non-elite mixed sport adolescent population, an area of sports injury research which requires further investigation [9].

Bodyweight also has a statistically significant association with injury severity with higher participant BW increasing the predicted injury severity (Table 3). However, it would be a concerning result to point towards greater BW as a direct risk factor for sports related injury. BW explained only 4% of the variance in injury severity for this unadjusted model and BMI was a non-significant variable. Bodyweight is not synonymous with body composition. This limits our ability to determine where specific components of body composition are associated with injury severity in adolescents. This highlights the need for further research into body composition as a risk factor for sports injury, as higher BMI has been identified as a potentially relevant risk factor [2, 36, 37].

The statistically significant association of posteromedial GYRO, posterolateral JERK and posteromedial GYRO SEN in predicting risk of time loss and non-time loss injuries in the unadjusted ordinal regression models show the advantages of using the IMU leveraged QYBT to assess dynamic balance. Particularly given that analogue reach distances did not show meaningful associations with injury severity. This highlights the ability of IMU’s to be utilised in dynamic balance assessment to gain more precise measures of COM control compared to simple reach distances and observing postural control more objectively for sports clinicians [5, 23]. The utilisation of IMU’s to improve dynamic balance and motor control assessment is supported in the literature which has been shown to detect both those at risk of injury in a sporting context and those still with postural control deficits post-concussion [5, 23, 38].

### Perspective

Dynamic balance performance, athletic exposures and BW represent three trackable and modifiable risk factors for injury in adolescent sports [2, 5]. These three factors should be considered by sports clinicians working with injured adolescents returning to sports as well as coaches within adolescent sports. Dynamic balance performance can impact the severity of sports related injury in an adolescent population but deficits in dynamic balance performance can be missed with some standard measures like the normalised reach distances as measured on the YBT [5, 23]. The utilisation of IMU technology to assess dynamic balance and lower limb motor control can give a more objective measure of COM control [5, 23]. This may indicate sub-optimal balance performance leading to risk of more severe injury that may be missed using standard methods like normalized reach distances [5, 23]. Athletic exposures should be monitored and kept consistent in this adolescent cohort as increases in athletic exposures appears to increase the risk of injury in the following week within this adolescent cohort [13].

### Limitations

A limitation of this study was the presence of missing data points from baseline measures as detailed in Table 2. These missing data points are missing at random due to equipment failure. These missing data points from baseline measures were repeated in the analysed repeated measures data set. To avoid introducing bias within the analysis no imputation was done and the analysis was conducted on the available data with missing data points excluded. This reduced the risk of introducing bias and means results were produced solely on observed data. Another limitation of this study was the overall response rate of 48% which is similar to other studies involving an adolescent sample [39, 40]. Lastly, our study had relatively small sample size and the included sports were majorly field hockey and netball for females and majorly rugby and Gaelic football for males. However, this may be representative of the general adolescent sports population in secondary schools on the Island of Ireland. Furthermore we are unaware if the student participants sought treatment for any of their sports injuries as we did not include this in the biweekly questionnaire in order to reduce burden of participation keeping the questionnaire as short as possible in order to maintain adherence. We accounted for any reduction in athletic exposures the week of an injury was reported by calculating athletic exposures as the rolling average weekly number of athletic exposures lagged by one week. Finally we were unable to capture body composition metrics beyond BMI and BW. We recommend that future research include more detailed body composition assessments to clarify these relationships.

## Conclusion

The results of this study demonstrate the association of athletic exposures and dynamic balance performance with sports related injury severity in a mixed sport adolescent cohort. The results also show the strong predictive power and association of increased athletic exposures with increased risk of more severe injury while slower COM control during dynamic balance assessment in the QYBT is significantly associated with more severe injury (e.g. increased risk of time loss injury compared to non-time loss injury). This study also highlighted that body composition (e.g. lean body mass) may be a risk factor for increased injury severity in adolescent sports. Dynamic balance performance and athletic exposures constitute trackable risk factors associated with sports injury in adolescent sports that can be modified to mitigate the risk and severity of injury.

## Supplementary Information

Supplementary Material 1.

Supplementary Material 2.

## Data Availability

The datasets used and/or analysed during the current study are available from the corresponding author on reasonable request.

## Abbreviations

BMI: Body mass index
BW: Bodyweight
CI: Confidence interval
COM: Centre of mass
GYRO: Gyroscopic magnitude root mean squared
GYRO: SEN Gyroscopic magnitude sample entropy
IMU: Inertial measurement unit
IQR: Interquartile Range
OR: Odds ratio
QYBT: Quantified Y-Balance Test
SD: Standard Deviation

## References

[1] McKay CD, Cumming SP, Blake T. Youth sport: Friend or Foe? Best Pract Res Clin Rheumatol. 2019;33(1):141–57. 31431268 10.1016/j.berh.2019.01.017

[2] Al-Qahtani MA, Allajhar MA, Alzahrani AA, Asiri MA, Alsalem AF, Alshahrani SA, et al. Sports-Related Injuries in Adolescent Athletes: A Systematic Review. Cureus. 2023;15(11):e49392. 38146581 10.7759/cureus.49392 PMC10749669

[3] Emmet D, Roberts J, Yao KV. Update on Preventing Overuse Injuries in Youth Athletes. Curr Phys Med Rehabilitation Rep. 2022;10(3):248–56.

[4] Sheehan N, Summersby R, Bleakley C, Caulfield B, Matthews M, Klempel N, et al. Adolescents’ experience with sports-related pain and injury: A systematic review of qualitative research. Phys Ther Sport. 2024;68:7–21. 38843686 10.1016/j.ptsp.2024.05.003

[5] Johnston W, O’Reilly M, Duignan C, Liston M, McLoughlin R, Coughlan GF, et al. Association of Dynamic Balance With Sports-Related Concussion: A Prospective Cohort Study. Am J Sports Med. 2019;47(1):197–205. 30501391 10.1177/0363546518812820

[6] Parry GN, Williams S, McKay CD, Johnson DJ, Bergeron MF, Cumming SP. Associations between growth, Maturation and Injury in Youth Athletes Engaged in Elite pathways: a Scoping Review. Br J Sports Med. 2024;58(17):bjsports-108233. 10.1136/bjsports-2024-108233 PMC11420720 39209526

[7] Sainsbury DA, Downs J, Netto K, McKenna LJ. Factors Associated With Sports Injuries in Adolescents Who Play Team Sports at a Nonelite Level: A Scoping Review. JOSPT Open. 2023;1(1):1–15.

[8] Hall ECR, Larruskain J, Gil SM, Lekue JA, Baumert P, Rienzi E, et al. Injury risk is greater in physically mature versus biologically younger male soccer players from academies in different countries. Phys Ther Sport. 2022;55:111–8. 35325670 10.1016/j.ptsp.2022.03.006

[9] Verstappen S, van Rijn RM, Cost R, Stubbe JH. The Association Between Training Load and Injury Risk in Elite Youth Soccer Players: a Systematic Review and Best Evidence Synthesis. Sports Med Open. 2021;7(1):6. 33428001 10.1186/s40798-020-00296-1 PMC7801562

[10] Impellizzeri FM, McCall A, Ward P, Bornn L, Coutts AJ. Training Load and Its Role in Injury Prevention, Part 2: Conceptual and Methodologic Pitfalls. J Athl Train. 2020;55(9):893–901. 32991699 10.4085/1062-6050-501-19 PMC7534938

[11] Chan CC, Yung PSH, Mok KM. The Relationship between Training Load and Injury Risk in Basketball: A Systematic Review. Healthcare. 2024;12(18):1829–9. 10.3390/healthcare12181829 PMC11431307 39337170

[12] Damsted C, Glad S, Nielsen RO, Sørensen H, Malisoux L. IS THERE EVIDENCE FOR AN ASSOCIATION BETWEEN CHANGES IN TRAINING LOAD AND RUNNING-RELATED INJURIES? A SYSTEMATIC REVIEW. Int J Sports Phys Ther. 2018;13(6):931–42. PMC6253751 30534459

[13] Mason J, Wellmann K, Groll A, Braumann KM, Junge A, Hollander K, et al. Game Exposure, Player Characteristics, and Neuromuscular Performance Influence Injury Risk in Professional and Youth Field Hockey Players. Orthop J Sports Med. 2021;9(4):2325967121995167. 33889643 10.1177/2325967121995167 PMC8033403

[14] Junge T, Runge L, Juul-Kristensen B, Wedderkopp N. Risk Factors for Knee Injuries in Children 8 to 15 Years: The CHAMPS Study DK. Med Sci Sports Exerc. 2016;48(4):655–62. 26559452 10.1249/MSS.0000000000000814

[15] Plisky P, Schwartkopf-Phifer K, Huebner B, Garner MB, Bullock G. Systematic Review and Meta-Analysis of the Y-Balance Test Lower Quarter: Reliability, Discriminant Validity, and Predictive Validity. Int J Sports Phys Ther. 2021;16(5):1190–209. 34631241 10.26603/001c.27634 PMC8486397

[16] Plisky PJ, Rauh MJ, Kaminski TW, Underwood FB. Star Excursion Balance Test as a predictor of lower extremity injury in high school basketball players. J Orthop Sports Phys Ther. 2006;36(12):911–9. 17193868 10.2519/jospt.2006.2244

[17] Quatman-Yates CC, Quatman CE, Meszaros AJ, Paterno MV, Hewett TE. A systematic review of sensorimotor function during adolescence: a developmental stage of increased motor awkwardness? Br J Sports Med. 2012;46(9):649–55. 21459874 10.1136/bjsm.2010.079616 PMC4157222

[18] Johnston W, O’Reilly M, Coughlan Garrett F, Caulfield B. Inertial Sensor Technology Can Capture Changes in Dynamic Balance Control during the Y Balance Test. Digit Biomark. 2018;1(2):106–17. 10.1159/000485470 PMC7015365 32095752

[19] Bahr R, Clarsen B, Derman W, Dvorak J, Emery CA, Finch CF, et al. International Olympic Committee consensus statement: methods for recording and reporting of epidemiological data on injury and illness in sport 2020 (including STROBE Extension for Sport Injury and Illness Surveillance (STROBE-SIIS)). Br J Sports Med. 2020;54(7):372–89. 32071062 10.1136/bjsports-2019-101969 PMC7146946

[20] von Elm E, Altman DG, Egger M, Pocock SJ, Gotzsche PC, Vandenbroucke JP, et al. The Strengthening the Reporting of Observational Studies in Epidemiology (STROBE) statement: guidelines for reporting observational studies. Prev Med. 2007;45(4):247–51. 17950122 10.1016/j.ypmed.2007.08.012

[21] Koziel SM, Malina RM. Modified Maturity Offset Prediction Equations: Validation in Independent Longitudinal Samples of Boys and Girls. Sports Med. 2018;48(1):221–36. 28608181 10.1007/s40279-017-0750-y PMC5752743

[22] van Melick N, Meddeler BM, Hoogeboom TJ, Nijhuis-van der Sanden MWG, van Cingel REH. How to determine leg dominance: The agreement between self-reported and observed performance in healthy adults. PLoS ONE. 2017;12(12):e0189876. 29287067 10.1371/journal.pone.0189876 PMC5747428

[23] Johnston W, Heiderscheit B, Sanfilippo J, Brooks MA, Caulfield B. Athletes with a concussion history in the last two years have impairments in dynamic balance performance. Scand J Med Sci Sports. 2020;30(8):1497–505. 32311175 10.1111/sms.13691

[24] Clarsen B, Bahr R, Myklebust G, Andersson SH, Docking SI, Drew M, et al. Improved reporting of overuse injuries and health problems in sport: an update of the Oslo Sport Trauma Research Center questionnaires. Br J Sports Med. 2020;54(7):390–6. 32060142 10.1136/bjsports-2019-101337

[25] Medina McKeon JM, McKeon PO, Nedimyer AK. Sports Injury Epidemiology: Foundation of Evidence of, by, and for Athletic Trainers. J Athl Train. 2021;56(7):606–15. 34280283 10.4085/1062-6050-625-20 PMC8293889

[26] Bahr R, Clarsen B, Ekstrand J. Why we should focus on the burden of injuries and illnesses, not just their incidence. Brit J Sports Med. 2017;52(16):1018–21. 10.1136/bjsports-2017-098160 29021247

[27] Ramdani S, Seigle B, Lagarde J, Bouchara F, Bernard PL. On the use of sample entropy to analyze human postural sway data. Med Eng Phys. 2009;31(8):1023–31. 19608447 10.1016/j.medengphy.2009.06.004

[28] Mancini M, Horak FB, Zampieri C, Carlson-Kuhta P, Nutt JG, Chiari L. Trunk accelerometry reveals postural instability in untreated Parkinson’s disease. Parkinsonism Relat Disord. 2011;17(7):557–62. 21641263 10.1016/j.parkreldis.2011.05.010 PMC5327861

[29] Melendez-Calderon A, Shirota C, Balasubramanian S. Estimating Movement Smoothness From Inertial Measurement Units. Front Bioeng Biotechnol. 2020;8:558771. 33520949 10.3389/fbioe.2020.558771 PMC7841375

[30] Chen H, Schall MC, Fethke NB. Gyroscope vector magnitude: A proposed method for measuring angular velocities. Appl Ergon. 2023;109:103981. 36739779 10.1016/j.apergo.2023.103981

[31] Cavanaugh JT, Guskiewicz KM, Giuliani C, Marshall S, Mercer VS, Stergiou N. Recovery of postural control after cerebral concussion: new insights using approximate entropy. J Athl Train. 2006;41(3):305–13. 17043699 PMC1569549

[32] Clarsen B, Myklebust G, Bahr R. Development and validation of a new method for the registration of overuse injuries in sports injury epidemiology: the Oslo Sports Trauma Research Centre (OSTRC) overuse injury questionnaire. Br J Sports Med. 2013;47(8):495–502. 23038786 10.1136/bjsports-2012-091524

[33] von Rosen P, Heijne A, Frohm A, Friden C, Kottorp A. High Injury Burden in Elite Adolescent Athletes: A 52-Week Prospective Study. J Athl Train. 2018;53(3):262–70. 29412695 10.4085/1062-6050-251-16 PMC5894377

[34] Moher D, Hopewell S, Schulz KF, Montori V, Gotzsche PC, Devereaux PJ, et al. CONSORT 2010 Explanation and Elaboration: Updated guidelines for reporting parallel group randomised trials. J Clin Epidemiol. 2010;63(8):e1–37. 20346624 10.1016/j.jclinepi.2010.03.004

[35] Fernández D, Estopañan M, Baumer B, Casals M. Reporting of Regression Models for Ordinal Responses in Sports Sciences Field: A Systematic Review. WIREs Comput Stat. 2025;17(1).

[36] Richmond SA, Nettel-Aguirre A, Doyle-Baker PK, Macpherson A, Emery CA. Examining Measures of Weight as Risk Factors for Sport-Related Injury in Adolescents. J Sports Med. 2016;2016:7316947. Available from: https://www.ncbi.nlm.nih.gov/pmc/articles/PMC4971326/. 10.1155/2016/7316947 PMC4971326 27525304

[37] Prieto-González P, Martínez-Castillo JL, Fernández-Galván LM, Casado A, Soporki S, Sánchez-Infante J. Epidemiology of Sports-Related Injuries and Associated Risk Factors in Adolescent Athletes: An Injury Surveillance. Int J Environ Res Public Health. 2021;18(9):4857. 10.3390/ijerph18094857 PMC8125505 34063226

[38] Johnston W, Davenport J, Connelly R, Caulfield B. Quantifying Y Balance Test performance with multiple and single inertial sensors. Annu Int Conf IEEE Eng Med Biol Soc. 2020;2020:4243–7. 33018933 10.1109/EMBC44109.2020.9176416

[39] Hausken-Sutter SE, Schubring A, Grau S, Af Gennas KB, Barker-Ruchti N. Methodological implications of adapting and applying a web-based questionnaire on health problems to adolescent football players. BMC Med Res Methodol. 2021;21(1):252. 34781894 10.1186/s12874-021-01406-7 PMC8594195

[40] Moseid CH, Myklebust G, Fagerland MW, Clarsen B, Bahr R. The prevalence and severity of health problems in youth elite sports: A 6-month prospective cohort study of 320 athletes. Scand J Med Sci Sports. 2018;28(4):1412–23. 29281145 10.1111/sms.13047

