# Supplementary material for: Risk factors associated with sports related injury severity in adolescents: a prospective study over a single season of sport
Source: BMC Sports Sci Med Rehabil. 2026 Apr 14;18:245. doi: 10.1186/s13102-026-01632-w (PMC13200426; doi:10.1186/s13102-026-01632-w)
Supplement: Supplementary file 1 — Supplementary Material 1. [file 13102_2026_1632_MOESM1_ESM.docx]

Appendix 1.

**Appendix 1. – Full Follow Up Questionnaire**

**App Based Questionnaire**

**1.  Have you had any difficulties participating in training and competition due to a sports related pain or an injury during the past 2 weeks?**

1. Full participation without  pain/injury
2. Full participation, but with  pain/injury
3. Reduced participation due to  pain/injury
4. Could not participate due to  pain/injury.

**2. To what extent have you modified your training or competition due to the sports related pain/injury over the past 2 weeks?**

1. No modification
2. To a minor extent
3. To a moderate extent
4. To a major extent

**3. To what extent has your sports related pain/injury affected your performance over the past 2 weeks?**

1. No effect
2. To a minor extent
3. To a moderate extent
4. To a major extent

**4. To what extent have you experienced pain from your sports related pain/injury over the past 2 weeks?**

1. No pain
2. Mild pain

Moderate Pain

Severe Pain

**5. Please indicate the body part most affected by your injury/pain?**

Head

Neck

Back

Shoulder

Chest

Wrist

Hand

Arm

Hip

Leg

Knee

Ankle

Foot

Other

**6. Over the past 2 weeks how many games/matches/races have you competed in?**

Options 0-15

**7. Over the past 2 weeks how many training sessions have you had on field/court/track/pool/or elsewhere in all your sports?**

Options 0-15

**8. Over the past 2 weeks how many gym based sessions have you had?**

Options 0-15
