# Supplementary material for: Risk factors associated with sports related injury severity in adolescents: a prospective study over a single season of sport
Source: BMC Sports Sci Med Rehabil. 2026 Apr 14;18:245. doi: 10.1186/s13102-026-01632-w (PMC13200426; doi:10.1186/s13102-026-01632-w)
Supplement: Supplementary file 2 — Supplementary Material 2. [file 13102_2026_1632_MOESM2_ESM.docx]

Appendix 2.

## QYBT Variable Formulas

Each of the QYBT outcome measures shown below were recorded in each of the three QYBT reach directions (see Figure 2): anterior, posteromedial and posterolateral.

**Jerk magnitude root mean squared** (JERK): JERK is calculated using the following formula:

$$Jerk Magnitude Root Mean Squared= \sqrt{\frac{1}{T}\int_{0}^{T} x^{2}\left( t \right)+y^{2}\left( t \right)+z^{2}\left( t \right)}dt$$

Where x(t), y(t), z(t) represent the jerk in the x, y and z axis respectively at time (t) of the time series signal. Τ represents the overall time period of the signal.

**Gyroscopic magnitude root mean squared** (GYRO): GYRO is calculated using the following formula:

$$Gyroscopic Magnitude Root Mean Squared= \sqrt{\frac{1}{T}\int_{0}^{T} x^{2}\left( t \right)+y^{2}\left( t \right)+z^{2}\left( t \right)}dt$$

Where x(t), y(t), z(t) represent the angular velocity in the sagittal, transverse and coronal planes respectively at time (t) of the time series signal. Τ represents the overall time period of the signal.

**Gyroscopic Magnitude Sample Entropy** (GYRO SEN): GYRO SEN of length N=[x_1_, x_2,_ x_3_,… x_N_] was calculated using an r value of 0.1 and an m value of 2 following standard GYRO SEN formulas from previous QYBT research [1, 2].

**Normalized Reach Distances (%)**: Analogue reach distances were also normalized relative to the participants leg length using the formula below as part of initial data processing [3]. The reach distance and QYBT inertial sensor measures were averaged across the three test trials to ensure reliability of test results.

(𝑅𝑒𝑎𝑐h 𝐷𝑖𝑠𝑡𝑎𝑛𝑐𝑒(𝑐𝑚)/𝐿𝑒𝑔 𝐿𝑒𝑛𝑔𝑡h(cm)) × (100/1)

# References

1. Johnston W, O'Reilly M, Duignan C, Liston M, McLoughlin R, Coughlan GF, et al. Association of Dynamic Balance With Sports-Related Concussion: A Prospective Cohort Study. Am J Sports Med. 2019;47(1):197-205.

2. Johnston W, Heiderscheit B, Sanfilippo J, Brooks MA, Caulfield B. Athletes with a concussion history in the last two years have impairments in dynamic balance performance. Scand J Med Sci Sports. 2020;30(8):1497-505.

3. Gribble PA, Hertel J, Plisky P. Using the Star Excursion Balance Test to assess dynamic postural-control deficits and outcomes in lower extremity injury: a literature and systematic review. J Athl Train. 2012;47(3):339-57.
